# The Presence of a Visual Dividing Line Increases Consumer Memory Through Attention Grabbing

**DOI:** 10.3389/fpsyg.2022.848471

**Published:** 2022-04-12

**Authors:** Jun Ouyang, Yanli Jia

**Affiliations:** ^1^Business School, Minnan Normal University, Zhangzhou, China; ^2^Department of Marketing, Xiamen University, Xiamen, China

**Keywords:** dividing line, consumer attention, eye-tracking, consumer memory, consumer behavior

## Abstract

Marketers often use a visual line to divide the product information on an advertisement into left-right (or top-bottom) segments for aesthetic or categorization purposes. The present research examined the effect of the dividing line on the consumer memory. Across three studies (including an eye-tracking study and a field one), we showed that the presence of a dividing line enhances consumers’ memory about the products displayed on the *left*/*top* of an advertisement. This effect occurs because the dividing line orients participants’ first eye fixation to the left/top area of the advertisement, such that their visual attention is largely restricted to that area and they could better remember the contents displayed on that area. The theoretical contributions and implications for marketers and consumers are discussed.

## Introduction

Rising levels of advertising competition have made it increasingly difficult to attract consumers’ attention and to establish strong memory about the advertised products (Burke and Srull, 1988; Pieters et al., 2002; Nguyen et al., 2018). At the same time, advertisement costs are rising; the average advertisement spending per capita in the United States has risen from 25 dollars shortly after the World War II to 200 dollars nowadays, two times as much as that in Canada, four times as that in United Kingdom and five times as that in France (Ries and Trout, 2001). This motivates advertisers and marketers to improve the effectiveness of advertisements. To achieve their goal, one viable way is to help consumers remember the key information (e.g., high-margin products or new arrivals) on the advertisement. Therefore, we are interested in how to facilitate consumers’ memory about this information through visual cues.

Previous research has suggested a number of factors that can improve consumers’ memory. For example, pictorial (vs. verbal) information leads to better consumers’ memory (Childers and Houston, 1984; Viswanathan et al., 2009). Moreover, consumers’ positive mood enhances their learning and recalling of product information relative to the neutral mood (Lee and Sternthal, 1999). Adding to these findings, we focus on one of the most fundamental visual design elements that has not been previously examined—a visual dividing line—and investigate its effects on consumers’ memory.

Dividing lines have been widely used in advertising/webpage designs. For example, the food items on a menu are usually divided into several different categories according to their flavors, series products are divided into different groups according to their functionalities, and even a news webpage is divided into different blocks for ease of reading. Despite the wide use of dividing lines, it remains unclear how the presence of a dividing line on an advertisement affects consumers’ memory and subsequently on their purchase behavior.

Our research addresses this gap. Specifically, we found that consumers can better remember the products displayed on the left/top half of the advertisement if a dividing line is presented (vs. absent) on the advertisement. Our research also shed light on the underlying mechanism, that is, the presence of a dividing line draws more consumers’ attention to the products on the left/top side, leading consumers to better remember these products, and subsequently increasing their actual purchase of these products.

## Theoretical Background

A dividing line refers to a visual boundary that dictates where things belong (Thorndyke, 1981; Burris and Branscombe, 2005; Cutright, 2012). Previous research on dividing lines focused on its functional benefits, such as serving as an aesthetic communication or categorizing different contents into different groups (Hartmann et al., 2008; Wen and Lurie, 2019). Beyond these functional benefits, our research focuses on its potential impact on guiding consumers’ attention allocation and subsequently on their memory, a topic that has been largely overlooked by prior research.

### Dividing Line in Attentional Allocation

Past research has demonstrated that the visual cues, such as shape, color, and size, can grab consumers’ attention (Huh, 1993; Folkes and Matta, 2004; Hagtvedt and Brasel, 2017). For instance, to the extent that a picture shown in a news report is large and vivid, recipients are likely to pay attention to it (Smith, 1991; Huh, 1993). Likewise, a product’s novel shape or bright color can also capture consumers’ attention (Folkes and Matta, 2004; Hagtvedt and Brasel, 2017). In line with these prior findings, we draw on the literature on dividing lines (e.g., Burris and Branscombe, 2005; Cutright, 2012; Hou et al., 2018) to propose that a dividing line can draw consumers’ attention to the left/top side of the line.

Specifically, a dividing line can orient people’s first eye fixation to the left/top area of an advertisement. Serving as a visual cue, a dividing line can make people be more aware of the “left-right” (or “top-bottom”) orientation of the advertisement than the other way around (Rayner, 1978, 2009; Bestgen et al., 2013). Once they are aware of the orientation, they would automatically locate their first fixation to the left/top area (Kaufman and Richards, 1969), because left-to-right (or top-to-bottom) eye movement is more consistent with their reading or writing habits (Lohse, 1997; Bulf et al., 2016; Zhang et al., 2019). The locations where people’s eyes first fixate, however, attract more attention than other locations (Lin et al., 2015). For examples, consumers tend to allocate more attention to the middle option that they first look at, than the right or the left options (Atalay et al., 2012). Likewise, they pay more attention to the product with a high color saturation that can quickly catch their eyes than that with a low saturation (Hagtvedt and Brasel, 2017). By contrast, if the dividing line is not available, people might first fixate their eyes anywhere depending on the visual salience of individual products and might pay attention to these products, not necessarily to the products displayed on the left/top (Yantis, 2005; Wedel and Pieters, 2008).

To summarize, we propose that a vertical/horizontal dividing line will restrict people’s attention to the products on the left/top of the advertisement rather than those on the right/bottom of it. If so, we propose that this biased attention allocation will lead to a better consumer memory of the products displayed on the left/top. We will elaborate on why this is so as below.

### Role of Attention in Consumer Memory

A number of prior research has suggested that multiple situational factors can impact consumers’ memory. For example, the stimuli presented in pictorial format are better recalled than those in verbal format (Childers and Houston, 1984; Viswanathan et al., 2009). Moreover, consumers can better recall the contents that arouse some emotion than those without emotion (Ambler and Burne, 1999; Guido et al., 2017) and they can better recall product information if the background music of an advertisement ends with a note in the chord of dominant tonality than that ends abruptly (Guido et al., 2016).

More germane to the present research, attention also serves as a key factor that affects consumer memory. The plethora of previous research has suggested that memory requires attention (Atkinson and Shiffrin, 1968; Baddeley, 1997; Styles, 2005). Consequently, attention distraction sabotages consumers’ memory (Craik et al., 1996; Mulligan, 1997, 1998). Following the same logic, we propose that if a dividing line increases consumers’ attention paid to the products presented on the left/top, they would better remember these products and therefore be more likely to purchase them when a dividing line was present than when it was not. Three studies examined these hypotheses.

## Study 1

Study 1 provided empirical evidence for our main hypothesis that a dividing line can improve consumers’ memory of the contents displayed on the left/top side of the advertisement.

### Method

Ninety-seven undergraduate students from a Chinese college (56.70% females; *M*_age_ = 23 ± 1.80 years) attended the study in lab for a small monetary reward. One participant who failed to follow the experimenter’s instruction was excluded,^1^ leaving 96 valid cases for data analysis. They were randomly assigned to cells of a three-condition (dividing line: vertical vs. horizontal vs. absent) between-subjects design.

Following a procedure used in previous research (Morrin and Ratneshwar, 2000; Viswanathan et al., 2009; Hartmann et al., 2013; Kelting and Rice, 2013), we told participants that the purpose of the study was to obtain feedback about how consumers would process the advertising information. Upon the pretense, participants were asked to evaluate a printed advertisement on which four toothpaste products under a fake brand name--‘‘COMVITA’’ were presented.^2^ The four toothpaste products were rotated and counterbalanced so that each product had an equal chance of appearing at the four locations on the advertisement (i.e., left top, left bottom, right top, and right bottom) across participants. In this way, we created a total of 24 versions of the advertisement in the study. The advertisement was evenly divided either by a vertical line, by a horizontal line, or not divided. See Supplementary Appendix A for one version of the advertisement as an example. As previous research on consumer memory (Edwards et al., 2002; Bradley et al., 2007; Kelting and Rice, 2013) often limits the time that participants can use to view the stimuli for memory test, we allowed participants to view the advertisement only for 25 s.

After browsing the advertisement, participants completed a series of memory tests. Their learning of the product information presented earlier was assessed by both a free recall measure and a recognition measure adopted from Richardson-Klavehn and Bjork (1988). For the free recall task, participants were asked to write down as many of the toothpastes’ names as they could remember seeing earlier. For the recognition task, participants were asked to indicate whether they recognized each of the twenty toothpastes’ attributes by circling either “Yes” or “No” (see Supplementary Appendix C). Finally, all participants were debriefed about the purpose of the study and were dismissed.

### Results and Discussion

#### Memory

We conducted two mixed ANOVA analyses to compare participants’ recall of product names in the line absent condition with the vertical condition and with the horizontal condition, respectively. First, to compare participants’ recall in the vertical with those in the line absent conditions, we excluded participants in the horizontal condition and conducted a mixed ANOVA on participants’ recall with the presence of vertical line as a between-subject factor and advertisement area (left vs. right side) as a within-subject factor. The results revealed a significant main effect of advertisement area [*F*(1,61) = 24.81, *p* < 0.001; partial η^2^ = 0.29] and a significant vertical line presence × advertisement area interaction [*F*(1,61) = 8.63, *p* < 0.01; partial η^2^ = 0.12]. Notably, the main effect of vertical line presence was not significant (*F* < 1), suggesting that the presence of vertical line did not enhance participants’ general recall of the advertisement information. Planned contrasts revealed the nature of the interaction. Participants can correctly recall more names on the left in the vertical line present condition (*M* = 1.25, *SD* = 0.72) than in the absent condition [*M* = 0.84, *SD* = 0.69; *F*(1,61) = 5.38, *p* < 0.05; partial η^2^ = 0.08], but there was no significant difference between two conditions for recalling names on the right-side [*M*_present_ = 0.38, *SD* = 0.49 vs. *M*_absent_ = 0.61, *SD* = 0.56; *F*(1,61) = 3.23, *p* = 0.08; partial η^2^ = 0.05]. Second, a similar mixed ANOVA with the presence of horizontal line as a between-subject factor and advertisement area (top vs. bottom side) as a within-subject factor was also conducted after excluding participants in the vertical condition. The results revealed a significant main effect of advertisement area [*F*(1,62) = 9.29, *p* < 0.01; partial η^2^ = 0.13], a non-significant main effect of horizontal line presence (*F* < 1), and more important a significant interaction [*F*(1,62) = 7.78, *p* < 0.01; partial η^2^ = 0.11]. Planned contrasts revealed that participants can correctly recall more names on the top in the horizontal line present condition (*M* = 1.12, *SD* = 0.70) than in the absent condition [*M* = 0.74, *SD* = 0.73; *F*(1,62) = 4.53, *p* < 0.05; partial η^2^ = 0.07], but they can recall fewer names on the bottom in the first condition (*M* = 0.39, *SD* = 0.56) than in the second condition [*M* = 0.71, *SD* = 0.53; *F*(1,62) = 5.41, *p* < 0.05; partial η^2^ = 0.08].

Moreover, the results of participants’ recognition test are consistent with those of free recall. Two mixed ANOVA analyses on the recognition of the product attributes were run. Specifically, after excluding participants in the horizontal condition, we conducted a mixed ANOVA with the presence of vertical line as a between-subject factor and advertisement area (left vs. right side) as a within-subject factor. The results revealed a significant main effect of advertisement area [*F*(1,61) = 10.01, *p* < 0.01; partial η^2^ = 0.14], a non-significant main effect of vertical line presence (*F* < 1) and more important a significant interaction [*F*(1,61) = 8.64, *p* < 0.01; partial η^2^ = 0.12]. Planned contrasts suggested that participants can correctly recognize more product attributes on the left-side in the vertical line present condition (*M* = 6.28, *SD* = 1.37) than in the absent condition [*M* = 5.42, *SD* = 1.29; *F*(1,61) = 6.61, *p* < 0.05; partial η^2^ = 0.10], but they can recognize fewer product attributes on the right-side in the first condition (*M* = 4.53, *SD* = 1.72) than in the second condition [*M* = 5.35, *SD* = 1.60; *F*(1,61) = 3.85, *p* = 0.05; partial η^2^ = 0.06]. A similar mixed ANOVA with the presence of horizontal line as a between-subject factor and advertisement area (top vs. bottom side) as a within-subject factor, after excluding participants in the vertical condition, revealed a significant main effect of advertisement area [*F*(1,62) = 23.04, *p* < 0.001; partial η^2^ = 0.27], a non-significant main effect of horizontal line presence (*F* < 1), and as expected a significant interaction [*F*(1,62) = 6.30, *p* < 0.05; partial η^2^ = 0.09]. Planned contrasts showed that participants can correctly recognize more product attributes on the top-side in the horizontal line present condition (*M* = 6.39, *SD* = 1.14) than in the absent condition [*M* = 5.71, *SD* = 1.40; *F*(1,62) = 4.63, *p* < 0.05; partial η^2^ = 0.07], but there was no significant difference between two conditions for recognizing product attributes on the bottom-side [*M*_present_ = 4.33, *SD* = 1.95 vs. *M*_absent_ = 5.06, *SD* = 1.46; *F*(1,62) = 2.86, *p* = 0.10; partial η^2^ = 0.04].

The results of Study 1 provided direct evidence for our hypothesis that a vertical/horizontal line that divides an advertisement improved participants’ memory of the product information displayed on the left/top of the advertisement. However, it remained unclear about the mechanism underlying this effect, which led to the design of the Study 2.

## Study 2

Study 2 provided process evidence in support of our theory that a vertical dividing line leads consumers to pay more attention to the left area of an advertisement and consequently to improve their memory of the product information presented on that area. Study 2 used eye-tracking data to examine this possibility.

### Method

Fifty-four undergraduate students (55.56% females; *M*_age_ = 21 ± 1.41 years) from a Chinese college participated the study in lab for a small monetary reward. They were randomly assigned to two cells: a vertical line present vs. absent. Each participant was run individually as in prior eye-track studies.

Participants were asked to seat in front of a 21-inch monitor (1280 × 1024 pixels) and read about a toothpaste advertisement (28.9 × 25.5 centimeters) similar to that used in Study 1. Half of them read the advertisement divided by a vertical dividing line, while the other half read the advertisement that was not divided. Before viewing the advertisement, however, participants’ seating position was adjusted and the eye-tracking device was calibrated by asking participants to focus on five red dots that were presented sequentially on different areas of the computer screen (for the mechanism behind the eye-tracking calibration, see Brisson et al., 2013). Participants were told that this calibration exercise was necessary to ensure that the quality of the video was good. After the calibration, participants were exposed to the advertisement and were asked to evaluate it. They were allowed to view the advertisement for 25 s, and their eye movements were recorded by a Tobii X2-60 eye tracker system with a 60 hertz sampling rate. They were then asked to complete the same memory tests used in Study 1 before they were debriefed about the purpose of the study.

The eye tracker recorded the time participants spent on the advertising page and the patterns of their eye movement. The eye movements could be categorized into areas of interest that were defined *a priori*. Two areas of interest were defined for each advertisement: the left area and the right area of the advertisement that are divided by the line. The initial landing position (i.e., the first fixation position; 1 = on the left, 0 = on the right) and the amount of time the eye dwelt on each area (i.e., eye fixations) were computed.

### Results and Discussion

#### Eye-Tracking Data

**FIGURE 1:**
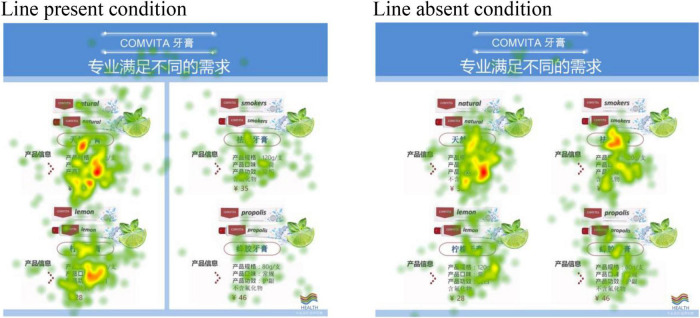
Eye-tracking heat map of Study 2.

Consistent with our theory, the analysis of the first fixation position revealed that participants were more likely to initially fixate on the left area of the advertisement if a dividing line was present (*M* = 77.78%) than if it was not [*M* = 51.85%; χ^2^(1) = 3.98, *p* < 0.05]. Moreover, a mixed ANOVA analysis on participants’ eye fixations with line presence as a between-subject factor and advertisement area (left vs. right side) as within-subjects factor revealed a significant main effect of advertisement area [*F*(1,52) = 46.49, *p* < 0.001; partial η^2^ = 0.47], a non-significant main effect of line presence (*F* < 1) and a significant interaction effect [*F*(1,52) = 36.82, *p* < 0.001; partial η^2^ = 0.42]. Planned contrasts showed that participants did spend more time dwelling on the left area of the advertisement when the dividing line was present (*M* = 8.92, *SD* = 3.94) than when it was not [*M* = 5.14, *SD* = 3.05; *F*(1,52) = 15.58, *p* < 0.001; partial η^2^ = 0.23]. By contrast, they spend less time dwelling on the right area in the first situation (*M* = 2.00, *SD* = 1.90) than in the second one [*M* = 4.73, *SD* = 2.57; *F*(1,52) = 19.63, *p* < 0.001; partial η^2^ = 0.27]. See Figure 1 for an example of the eye-tracking map.

#### Memory

The basic effect of the dividing line on participants’ recall of the product information was replicated in the study. A mixed ANOVA analysis of participants’ performance on the free recall test with line presence as a between-subject factor and advertisement area (left vs. right side) as a within-subject factor revealed a significant main effect of advertisement area [*F*(1,52) = 14.83, *p* < 0.001; partial η^2^ = 0.22], a non-significant main effect of line presence (*F* < 1), and more important a significant interaction [*F*(1,52) = 8.97, *p* < 0.01; partial η^2^ = 0.15]. Planned contrasts showed that participants can correctly recall more product names displayed on the left area of the advertisement in the line present condition (*M* = 1.33, *SD* = 0.83) than in the line absent condition [*M* = 0.93, *SD* = 0.68; *F*(1,52) = 3.90, *p* = 0.05; partial η^2^ = 0.07], but they can recall fewer product names displayed on the right area in the first condition (*M* = 0.44, *SD* = 0.64) than in the second condition [*M* = 0.81, *SD* = 0.68; *F*(1,52) = 4.24, *p* < 0.05; partial η^2^ = 0.08].

Moreover, a similar mixed ANOVA analysis on participants’ performance on the recognition test revealed a significant main effect of advertisement area [*F*(1,52) = 5.13, *p* < 0.05; partial η^2^ = 0.09], a non-significant main effect of line presence (*F* < 1), and as expected a significant interaction [*F*(1,52) = 11.31, *p* < 0.01; partial η^2^ = 0.18]. Planned contrasts indicated that participants can correctly recognize more left-sided product attributes in the line present condition (*M* = 6.81, *SD* = 1.11) than in the line absent condition [*M* = 5.85, *SD* = 1.26; *F*(1,52) = 8.86, *p* < 0.01; partial η^2^ = 0.15]. However, they can recognize fewer right-sided product attributes in the first condition (*M* = 5.30, *SD* = 1.46) than in the second condition [*M* = 6.15, *SD* = 1.54; *F*(1,52) = 4.35, *p* < 0.05; partial η^2^ = 0.08].

#### Mediation Analysis

To reiterate, we predicted that a dividing line would draw participants’ attention more to the left area of the advertisement, subsequently improve their memory of the information presented on this area. Therefore, we used the time participants fixate on the left area as an index of attentional bias. We conducted two independent bootstrapping analyses (Hayes, 2013; Model 4 in PROCESS) to examine the mediating role of participants’ attentional bias. Prior research (Zhao et al., 2010) suggests that although Baron and Kenny’s (1986) Sobel *z*-test has been widely used for establishing mediation, bootstrapping is almost always more powerful. Bootstrapping involves the repeated extraction of samples from the data set, in which we used 5,000 resamples, and the estimation of the indirect effect in the resampled dataset. First, we conducted a bootstrapping analysis with line condition as the independent variable and participants’ performance on the free recall test as the dependent variable yielded a significant indirect effect of the attentional bias (*B* = 0.30, *SE* = 0.13, 95% CI = 0.09–0.58), while the direct effect of line condition became non-significant (*B* = 0.10, *SE* = 0.22, 95% CI = −0.34–0.55). The same bootstrapping procedure using line condition as the independent variable and participants’ performance on the recognition test as the dependent variable also supported a significant indirect effect of the attentional bias (*B* = 0.64, *SE* = 0.22, 95% CI = 0.25–1.13), while the direct effect of line condition was not significant anymore (*B* = 0.32, *SE* = 0.32, 95% CI = −0.33--0.96).^3^

In conclusion, the results of Study 2 replicated those of Study 1. Moreover, using eye-tracking data, Study 2 provided empirical evidence that a dividing line directed participants’ attention more to the left area of the advertisement and facilitated their memory of the information presented on this area.

## Study 3

Because consumers’ memory positively influences their purchase (Lynch and Srull, 1982; Pechmann and Stewart, 1990; Coates et al., 2006), our findings imply that the presence of a dividing line should nudge consumer’ actual purchase of the products displayed on the left/top of an advertisement through the improved memory of the product information. To examine whether this is the case in a real-world setting, we conducted a field study at a local-product shop called “CREATIVE” over the course of two consecutive weeks (March 29–April 11, 2021). CREATIVE is located in a famous tourist spot in south China that mainly targets the tourists who visit the spot. To this end, there are very few repeat customers in the shop so that we can well control consumers’ prior purchase experience.

We implemented the manipulation of the dividing line on an advertisement shown at the entrance of the shop. Thus, when consumers visit the shop, they can view the advertisement as long as they want.

### Method

We selected one type of olive snack food with four different flavors to display on the advertisement.^4^ Each product was presented with a picture along with a short description of product features. The dividing line present vs. absent condition was switched every one week. Specifically, the four products were divided by a vertical line in the first week, but were not divided by the line in the week that followed (see Supplementary Appendix B).

In the procedure of data collection, we focused only on the consumers who visited the shop by themselves (rather than those who shopped together with friends) and who stopped to view the advertisement. Our experimenter recorded such consumers and tracked their subsequent purchase behaviors out of their awareness. Specifically, when such consumers made a purchase of the four snack food products shown on the advertisement, our experimenter recorded their purchase and invited them to fill in a simple survey ostensibly for improving the store services. On this pretense, we tested consumers’ recognition of the product features showed earlier on the advertisement. Finally, these consumers were debriefed about the purpose of the study and were asked to indicate whether they agreed to take part in the study. All of them agreed to participate.

To attract consumers’ purchase of the four products and increase the efficiency of our data collection, we launched a buy-2-get-1-free promotion. We in total recorded 215 consumers who viewed the advertisement (99 in the line present condition and 116 in the line absent condition). A total of 17 consumers did not make any purchase, leaving 198 consumers’ purchasing data for analysis (92 in the line present condition and 106 in the line absent condition).

### Results and Discussion

#### Amount of Purchase

A mixed ANOVA analysis of the amount of purchase (i.e., the amount of money participants spent on the target products) with dividing line (absent vs. present) as the between-subjects factor and advertisement area (left vs. right side) as a within-subject factor revealed a significant main effect of advertisement area [*F*(1,196) = 9.03, *p* < 0.01; partial η^2^ = 0.04], a non-significant main effect of dividing line [*F*(1,196) = 1.68, *p* = 0.20], and more important a significant interaction [*F*(1,196) = 21.44, *p* < 0.001; partial η^2^ = 0.10]. Planned contrasts revealed that consumers spent more on the products displayed on the left of advertisement when a vertical dividing line was present (*M* = 15.03, *SD* = 6.19) than when it was not [*M* = 12.12, *SD* = 6.78; *F*(1,196) = 9.77, *p* < 0.01; partial η^2^ = 0.05], but they spent less on the products displayed on the right of the advertisement in the first condition (*M* = 8.80, *SD* = 7.83) than in the second condition [*M* = 13.45, *SD* = 8.54; *F*(1,196) = 15.73, *p* < 0.001; partial η^2^ = 0.07].

#### Number of Purchased Items

A similar mixed ANOVA analysis of the number of purchased items revealed a significant main effect of advertisement area [*F*(1,196) = 9.03, *p* < 0.01; partial η^2^ = 0.04], a non-significant main effect of dividing line [*F*(1,196) = 1.68, *p* = 0.20], and a significant interaction [*F*(1,196) = 21.44, *p* < 0.001; partial η^2^ = 0.10]. Planned contrasts revealed that consumers purchased more product items displayed on the left side in the vertical dividing line present condition (*M* = 1.39, *SD* = 0.57) than in the line absent condition [*M* = 1.12, *SD* = 0.63; *F*(1,196) = 9.77, *p* < 0.01; partial η^2^ = 0.05], but they purchased fewer product items displayed on the right side in the first condition (*M* = 0.82, *SD* = 0.73) than in the second condition [*M* = 1.25, *SD* = 0.79; *F*(1,196) = 15.73, *p* < 0.001; partial η^2^ = 0.07].

#### Memory

A similar mixed ANOVA analysis of consumers’ recognition of the product features revealed a significant main effect of advertisement area [*F*(1,196) = 16.54, *p* < 0.001; partial η^2^ = 0.08], a non-significant main effect of dividing line (*F* < 1), and a significant interaction effect [*F*(1,196) = 13.73, *p* < 0.001; partial η^2^ = 0.07]. Planned contrasts showed that while consumers can better recognize the product features displayed on the left area in the line present condition (*M* = 1.40, *SD* = 0.70) than in the line absent condition [*M* = 1.07, *SD* = 0.83; *F*(1,196) = 9.35, *p* < 0.01; partial η^2^ = 0.05], they recognized fewer product features displayed on the right area in the first condition (*M* = 0.79, *SD* = 0.67) than in the second condition [*M* = 1.04, *SD* = 0.80; *F*(1,196) = 5.29, *p* < 0.05; partial η^2^ = 0.03].

#### Mediation Analysis

We conducted a bootstrapping analysis (Hayes, 2013; Model 4 in PROCESS) with line condition as the independent variable, amount of purchase as the dependent variable and memory as the mediator. The results suggested a significant indirect effect of memory at the 90% confidence interval (*B* = 0.39, *SE* = 0.26, 90% CI = 0.03–0.87) but a non-significant indirect effect at the 95% confidence interval (95% CI = −0.02--0.96), while the direct path from dividing line to amount of purchase remained significant (*B* = 2.51, *SE* = 0.94, 95% CI = 0.65--4.37).^5^ Notably, replacing the amount of purchase with number of purchased items in the above bootstrapping procedures did not change the results of our mediation analyses.

The results of this field study offered a real-world validation of the proposed effect by showing that a dividing line improved consumers’ memory of the products displayed on the left and subsequently increased their actual purchase of these products.

## Discussion

Through three experimental studies, this research provided robust evidence that participants can better remember the products displayed on the left/top half of the advertisement when there was a dividing line on the advertisement than when there was not. This effect occurred because the dividing line drew participants’ attention to the left/top area of the advertisement, leading them to better remember the products displayed on this area and to purchase the products.

The present research advances our knowledge in three ways. First, our findings extend previous research on dividing lines that focused either on the aesthetic communications or on categorization functions (Dimov, 2014; Wen and Lurie, 2019; Stanischewski et al., 2020). To our knowledge, very little research studied the impact of a dividing line on consumer memory. Our research indicated that a dividing line can improve consumers’ memory as a result of the attention-distributing role played by the line. Moreover, our research adds to prior research on consumer attention. While prior research focuses mainly on how the primary features of a stimulus such as color (bright vs. dim), size (big vs. small), or shape (regular vs. irregular) can draw consumer attention (Huh, 1993; Folkes and Matta, 2004; Hagtvedt and Brasel, 2017), our research focused on a design element of the stimulus that is totally irrelevant to its primary features and showed that a dividing line can orient consumer’s first fixation and distribute consumers’ attention disproportionately to the different segments of the stimulus. Finally, our research contributes to prior literature on consumer memory. Prior research has explored multiple factors that contribute to consumer memory, including picture vs. verbal presentation of an advertisement, background music when viewing the advertisement or viewers’ positive vs. negative affective states (Childers and Houston, 1984; Lee and Sternthal, 1999; Guido et al., 2016). Most of the research focuses on consumers’ memory toward a specific stimulus as a whole. The present research differs from prior research in that it documents that a dividing line can bias consumers’ attention to the different parts of a stimulus and increase their memory to some parts but decrease their attention to other parts, thus paving a way for future research in this domain.

From a managerial standpoint, our findings are meaningful since they show how a simple design element–a dividing line–affect consumer memory. In practice, although the marketers convey various information to consumers, they place different importance to the information. That said, they concern how to “pop out” the key product information (e.g., high-margin, new-arrived or trending products information) and enhance consumers’ memory of the information. In this case, it will be beneficial for marketers to use a vertical (or horizontal) line to divide an advertisement and present the key information on the left (or top) half of the advertisement. Moreover, as a dividing line serves to promote consumers’ memory of these products, marketers might be better by directly nudging consumers’ purchase from these products.

## Data Availability Statement

The raw data supporting the conclusions of this article will be made available by the authors, without undue reservation.

## Ethics Statement

The studies involving human participants were reviewed and approved by the Party Committee of Xiamen University. The patients/participants provided their written informed consent to participate in this study.

## Author Contributions

JO: conceptualization, methodology, data curation, writing–original draft, funding acquisition, and investigation. YJ: conceptualization, methodology, data curation, writing–original draft, funding acquisition, and supervision. Both authors contributed to the article and approved the submitted version.

## Conflict of Interest

The authors declare that the research was conducted in the absence of any commercial or financial relationships that could be construed as a potential conflict of interest.

## Publisher’s Note

All claims expressed in this article are solely those of the authors and do not necessarily represent those of their affiliated organizations, or those of the publisher, the editors and the reviewers. Any product that may be evaluated in this article, or claim that may be made by its manufacturer, is not guaranteed or endorsed by the publisher.

## Footnotes

1 The participant tried to go back to view the advertisement when doing the memory test.

2 A between-subjects pretest using 134 participants from the same population of the main study revealed that there were no significant differences among the four products in attractiveness (*F* < 1) and ease of remembering (*F* < 1).

3 The mediation analyses were run with participants’ performance on free recall test as the dependent variables or with participants’ performance on recognition test as the dependent variables, respectively. A first set of independently regressions revealed that the line condition significantly affected the performance on free recall test [β = 0.41, *t*(52) = 2.23, *p* = 0.05] and the performance on recognition test [β = 0.96, *t*(52) = 2.98, *p* < 0.05]. A second regression demonstrated that the line condition significantly affected the mediator (i.e., attentional bias) [β = 3.79, *t*(52) = 3.95, *p* < 0.001]. Finally, another set of independently conducted regressions, in which the line condition and the mediator were entered simultaneously, showed that the mediator significantly affected the performance on free recall test [β = 0.08, *t*(52) = 2.87, *p* < 0.01] and the performance on recognition test [β = 0.17, *t*(52) = 4.17, *p* < 0.001], whereas the effect of the line condition diminished significantly in the case of the performance on free recall test [β = 0.10, *t*(52) = 0.47, *p* = 0.64] and the performance on recognition test [β = 0.32, *t*(52) = 0.99, *p* = 0.33]. A set of Sobel tests indicated the attentional bias mediated the effect of dividing line in all two memory tasks: the free recall test (*z* = 2.32, *p* < 0.05) and the recognition test (*z* = 2.87, *p* < 0.01).

4 A within-subjects pretest consisting of 29 consumers from the same population of the main study showed that there were no significant differences among the four products in attractiveness (*F* < 1) and ease of remembering [*F*(1,28) = 1.07, *p* = 0.37].

5 A Sobel test was run to test the mediating role of memory. A first regression demonstrated that line condition had a significant effect on amount of purchase [β = 2.90, *t*(196) = 3.13, *p* < 0.01]. A second regression demonstrated that line condition had a significant effect on memory [β = 0.34, *t*(196) = 3.06, *p* < 0.01]. Finally, entering the memory and line condition in a regression showed that memory had a significant effect on amount of purchase [β = 1.17, *t*(196) = 1.96, *p* = 0.05], whereas the effect of dividing line on amount of purchase was still significant [β = 2.51, *t*(196) = 2.66, *p* < 0.01]. A Sobel test confirmed the pattern of results in the text (*z* = 1.65, *p* = 0.09).

## References

[B1] Ambler T. Burne T. (1999). The impact of affect on memory of advertising. *J. Adv. Res.* 39 25–34.

[B2] Atalay A. S. Bodur H. O. Rasolofoarison D. (2012). Shining in the center: central gaze cascade effect on product choice. *J. Consum. Res.* 39 848–866. 10.1086/665984

[B3] Atkinson R. C. Shiffrin R. M. (1968). “Human memory: a proposed system and its control processes,” in *Psychology of Learning and Motivation*, Vol. 2 eds Spence K. W. Spence J. T. (Amsterdam: Elsevier), 89–195. 10.1016/s0079-7421(08)60422-3

[B4] Baddeley A. D. (1997). *Human Memory: Theory and Practice.* London: Psychology press.

[B5] Baron R. M. Kenny D. A. (1986). The moderatormediator variable distinction in social psychological research: Conceptual, strategic, and statistical considerations. *J. Pers. Soc. Psychol.*, 51, 1173–1182. 3806354 10.1037//0022-3514.51.6.1173

[B6] Bestgen A. Edler D. Dickmann F. Kuchinke L. (2013). “Grid or no grid: distance distortion in recognizing spatial information from complex cartographic maps” in *Proceedings of the Annual Meeting of the Cognitive Science Society*, Berlin.

[B7] Bradley S. D. Angelini J. R. Lee S. (2007). Psychophysiological and memory effects of negative political ads: aversive, arousing, and well remembered. *J. Adv.* 36 115–127. 10.2753/joa0091-3367360409

[B8] Brisson J. Mainville M. Mailloux D. Beaulieu C. Serres J. Sirois S. (2013). Pupil diameter measurement errors as a function of gaze direction in corneal reflection eyetrackers. *Behav. Res. Methods* 45 1322–1331. 10.3758/s13428-013-0327-0 23468182

[B9] Bulf H. de Hevia M. D. Macchi Cassia V. (2016). Small on the left, large on the right: numbers orient visual attention onto space in preverbal infants. *Dev. Sci.* 19 394–401. 10.1111/desc.12315 26074348

[B10] Burke R. R. Srull T. K. (1988). Competitive interference and consumer memory for advertising. *J. Consum. Res.* 15 55–68. 10.1086/209145

[B11] Burris C. T. Branscombe N. R. (2005). Distorted distance estimation induced by a self-relevant national boundary. *J. Exp. Soc. Psychol.* 41 305–312. 10.1016/j.jesp.2004.06.012

[B12] Childers T. L. Houston M. J. (1984). Conditions for a picture-superiority effect on consumer memory. *J. Consum. Res.* 11 643–654. 10.1086/209001

[B13] Coates S. L. Butler L. T. Berry D. C. (2006). Implicit memory and consumer choice: the mediating role of brand familiarity. *Appl. Cogn. Psychol.* 20 1101–1116. 10.1002/acp.1262

[B14] Craik F. I. Govoni R. Naveh-Benjamin M. Anderson N. D. (1996). The effects of divided attention on encoding and retrieval processes in human memory. *J. Exp. Psychol. Gen.* 125 159–180. 10.1037/0096-3445.125.2.159 8683192

[B15] Cutright K. M. (2012). The beauty of boundaries: when and why we seek structure in consumption. *J. Consum. Res.* 38 775–790. 10.1086/661563

[B16] Dimov B. C. (2014). Perception of visual art element line on fine art works with pupils from I to VI grade. *Eur. Sci. J.* 1 575–579.

[B17] Edwards S. M. Li H. Lee J.-H. (2002). Forced exposure and psychological reactance: antecedents and consequences of the perceived intrusiveness of pop-up ads. *J. Adv.* 31 83–95. 10.1080/00913367.2002.10673678

[B18] Folkes V. Matta S. (2004). The effect of package shape on consumers’ judgments of product volume: attention as a mental contaminant. *J. Consum. Res.* 31 390–401. 10.1086/422117

[B19] Guido G. Peluso A. M. Mileti A. Capestro M. Cambò L. Pisanello P. (2016). Effects of background music endings on consumer memory in advertising. *Int. J. Adv.* 35 504–518. 10.1080/02650487.2015.1037233

[B20] Guido G. Pichierri M. Pino G. (2017). Place the good after the bad: effects of emotional shifts on consumer memory. *Mark. Lett.* 29 49–60. 10.1007/s11002-017-9439-0

[B21] Hagtvedt H. Brasel S. A. (2017). Color saturation increases perceived product size. *J. Consum. Res.* 44 396–413.

[B22] Hartmann J. Sutcliffe A. De Angeli A. (2008). Towards a theory of user judgment of aesthetics and user interface quality. *ACM Trans. Comput. Hum. Interact.* 15:15.

[B23] Hartmann P. Apaolaza V. Alija P. (2013). Nature imagery in advertising: attention restoration and memory effects. *Int. J. Adv.* 32 183–210. 10.2501/ija-32-2-183-210

[B24] Hayes A. F. (2013). Introduction to mediation, moderation, and conditional process analysis: a regression-based approach. *J. Educ. Meas.* 51 335–337. 10.1111/jedm.12050

[B25] Hou Y. Sun Y. Wan L. C. Wan Y. (2018). How can psychological contagion effect be attenuated? The role of boundary effect on menu design. *J. Hosp. Tour. Res.* 42 606–626. 10.1177/1096348015619410

[B26] Huh H.-J. L. (1993). The effect of newspaper picture size on readers’ attention, recall, and comprehension of stories. *High. Educ.* 43 3–40.

[B27] Kaufman L. Richards W. (1969). Spontaneous fixation tendencies for visual forms. *Percept. Psychophys.* 5 85–88. 10.3758/bf03210527

[B28] Kelting K. Rice D. H. (2013). Should we hire David Beckham to endorse our brand? Contextual interference and consumer memory for brands in a celebrity’s endorsement portfolio. *Psychol. Mark.* 30 602–613. 10.1002/mar.20631

[B29] Lee A. Y. Sternthal B. (1999). The effects of positive mood on memory. *J. Consum. Res.* 26 115–127. 10.1086/209554

[B30] Lin H. Lin W. Tsai W. C. Hsieh Y. C. Wu F. G. (2015). How different presentation modes of graphical icons affect viewers’ first fixation and attention. *Paper presented at the International Conference on Universal Access in Human-Computer Interaction* (Cham: Springer).

[B31] Lohse G. L. (1997). Consumer eye movement patterns on yellow pages advertising. *J. Adv.* 26 61–73. 10.1080/00913367.1997.10673518

[B32] Lynch J. G. Jr. Srull T. K. (1982). Memory and attentional factors in consumer choice: concepts and research methods. *J. Consum. Res.* 9 18–37. 10.1086/208893

[B33] Morrin M. Ratneshwar S. (2000). The impact of ambient scent on evaluation, attention, and memory for familiar and unfamiliar brands. *J. Bus. Res.* 49 157–165. 10.1016/s0148-2963(99)00006-5

[B34] Mulligan N. W. (1997). Attention and implicit memory tests: the effects of varying attentional load on conceptual priming. *Mem. Cogn.* 25 11–17. 10.3758/bf03197281 9046866

[B35] Mulligan N. W. (1998). The role of attention during encoding in implicit and explicit memory. *J. Exp. Psychol. Learn. Mem. Cognn.* 24:27. 10.1037//0278-7393.24.1.27 9438952

[B36] Nguyen C. Romaniuk J. Faulkner M. Cohen J. (2018). Are two brands better than one? Investigating the effects of co-branding in advertising on audience memory. *Mark. Lett.* 29 37–48. 10.1007/s11002-017-9444-3

[B37] Pechmann C. Stewart D. W. (1990). The effects of comparative advertising on attention, memory, and purchase intentions. *J. Consum. Res.* 17 180–191. 10.1086/208548

[B38] Pieters R. Warlop L. Wedel M. (2002). Breaking through the clutter: benefits of advertisement originality and familiarity for brand attention and memory. *Manage. Sci.* 48 765–781. 10.1287/mnsc.48.6.765.192 19642375

[B39] Rayner K. (1978). Eye movements in reading and information processing. *Psychol. Bull.* 85 618–660. 10.1037/0033-2909.85.3.618 353867

[B40] Rayner K. (2009). The 35th Sir Frederick Bartlett lecture: eye movements and attention in reading, scene perception, and visual search. *Q. J. Exp. Psychol.* 62 1457–1506. 10.1080/17470210902816461 19449261

[B41] Richardson-Klavehn A. Bjork R. A. (1988). Measures of memory. *Annu. Rev. Psychol.* 39 475–543.

[B42] Ries A. Trout J. (2001). *Positioning: The Battle for Your Mind.* New York, NY: McGraw Hill.

[B43] Smith R. A. (1991). The effects of visual and verbal advertising information on consumers’ inferences. *J. Adv.* 20 13–24. 10.1080/00913367.1991.10673351

[B44] Stanischewski S. Altmann C. S. Brachmann A. Redies C. (2020). Aesthetic perception of line patterns: effect of edge-orientation entropy and curvilinear shape. *i-Perception* 11 1–20. 10.1177/2041669520950749 33062240 PMC7533941

[B45] Styles E. A. (2005). *Attention, Perception and Memory: An Integrated Introduction.* New York, NY: Psychology Press.

[B46] Thorndyke P. W. (1981). Distance estimation from cognitive maps. *Cogn. Psychol.* 13 526–550. 10.1016/0010-0285(81)90019-0

[B47] Viswanathan M. Torelli C. J. Xia L. Gau R. (2009). Understanding the influence of literacy on consumer memory: the role of pictorial elements. *J. Consum. Psychol.* 19 389–402. 10.1016/j.jcps.2009.04.002

[B48] Wedel M. Pieters R. (2008). *Visual Marketing: From Attention to Action.* New York, NY: Psychology Press.

[B49] Wen N. Lurie N. H. (2019). More than aesthetic: visual boundaries and perceived variety. *J. Retail.* 95 86–98. 10.1016/j.jretai.2019.03.001

[B50] Yantis S. (2005). How visual salience wins the battle for awareness. *Nat. Neurosci.* 8 975–977. 10.1038/nn0805-975 16047021

[B51] Zhang Y. Kwak H. Jeong H. Puzakova M. (2019). Facing the “Right” Side? The effect of product facing direction. *J. Adv.* 48 153–166. 10.1080/00913367.2018.1503576

[B52] Zhao X. Lynch J. G. Jr. Chen Q. (2010). Reconsidering Baron and Kenny: myths and truths about mediation analysis. *J. Consum. Res.* 37 197–206. 10.1086/651257

