# Supplementary material for: The Presence of a Visual Dividing Line Increases Consumer Memory Through Attention Grabbing
Source: Front Psychol. 2022 Apr 12;13:848471. doi: 10.3389/fpsyg.2022.848471 (PMC9039128; doi:10.3389/fpsyg.2022.848471)
Supplement: Supplementary file 1 [file Data_Sheet_1.pdf]

Appendix A. Experimental Materials Used in Study 1 & 2.

Vertical dividing line present	Horizontal dividing line present
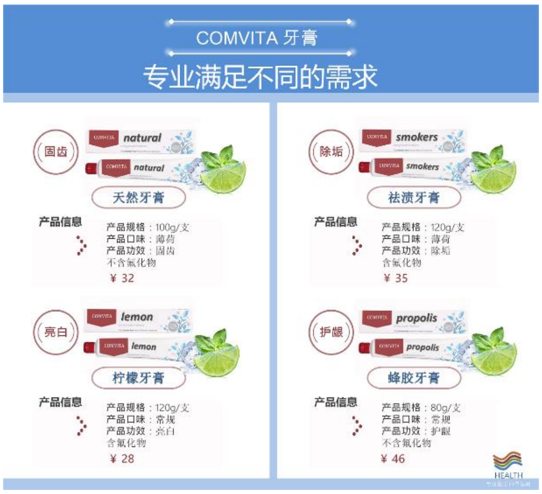	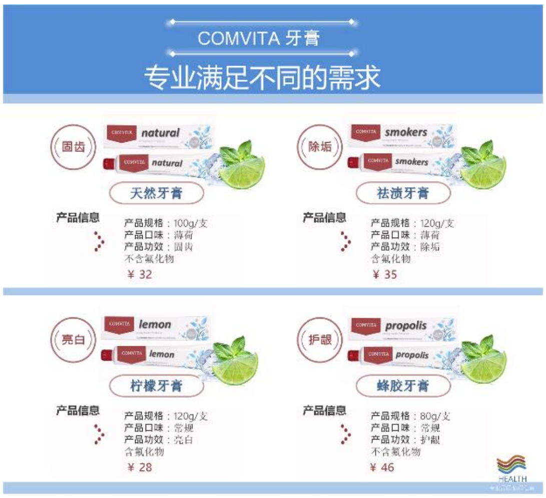

Dividing line absent

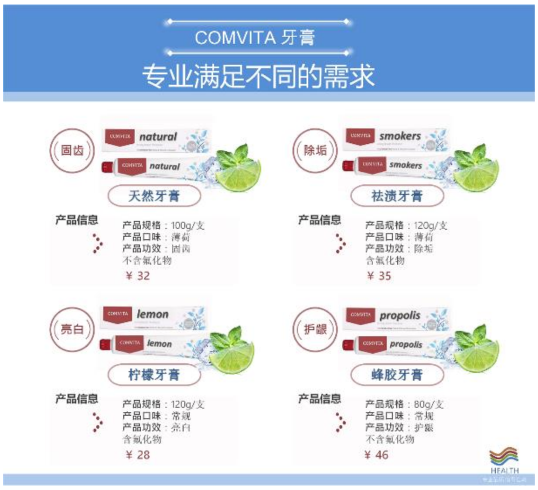

English translations:

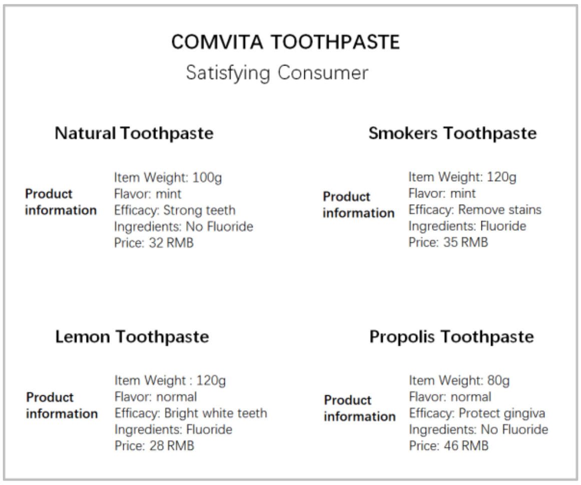

Appendix B. Experimental Materials Used in Study 3.

Line present condition	Line absent condition

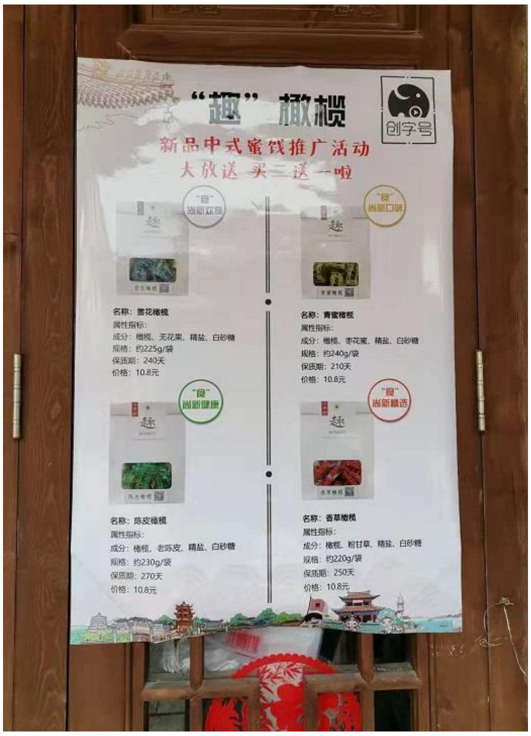

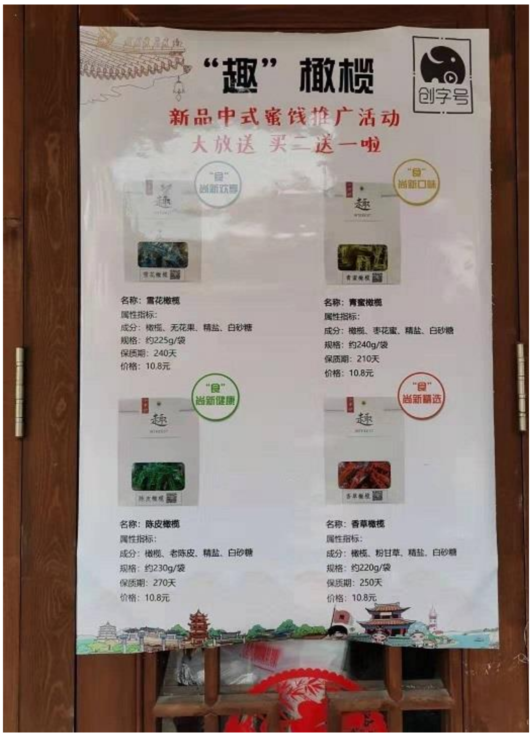

English translations:

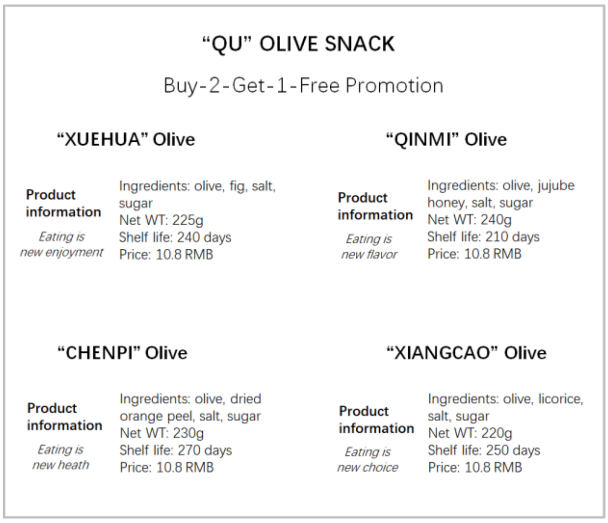

Appendix C. Toothpaste Attributes Tested in Recognition Test in Study 1 & 2.

		请判断对错			请判断对错
天然牙膏			祛渍牙膏		
1.	天然牙膏的产品规格是 120g/支。	YES NO	11.	祛渍牙膏的产品规格是 120g/支。	YES NO
2.	天然牙膏的产品口味是薄荷。	YES NO	12.	祛渍牙膏的产品口味是薄荷。	YES NO
3.	天然牙膏的产品功效是固齿。	YES NO	13.	祛渍牙膏的产品功效是亮白。	YES NO
4	天然牙膏含氟化物。	YES NO	14.	祛渍牙膏含氟化物。	YES NO
5.	天然牙膏的价格是 32 元。	YES NO	15.	祛渍牙膏的价格是 32 元。	YES NO
柠檬牙膏			蜂胶牙膏		
6.	柠檬牙膏的产品规格是 120 g/支。	YES NO	16.	蜂胶牙膏的产品规格是 100g/支。	YES NO
7.	柠檬牙膏的产品口味是常规。	YES NO	17.	蜂胶牙膏的产品口味是常规。	YES NO
8.	柠檬牙膏的产品功效是除垢。	YES NO	18.	蜂胶牙膏的产品功效是护龈。	YES NO
9.	柠檬牙膏不含氟化物。	YES NO	19.	蜂胶牙膏不含氟化物。	YES NO
10.	柠檬牙膏的价格是 28 元。	YES NO	20.	蜂胶牙膏的价格是 48 元。	YES NO
